# A Radiomic Nomogram for the Ultrasound-Based Evaluation of Extrathyroidal Extension in Papillary Thyroid Carcinoma

**DOI:** 10.3389/fonc.2021.625646

**Published:** 2021-03-04

**Authors:** Xian Wang, Enock Adjei Agyekum, Yongzhen Ren, Jin Zhang, Qing Zhang, Hui Sun, Guoliang Zhang, Feiju Xu, Xiangshu Bo, Wenzhi Lv, Shudong Hu, Xiaoqin Qian

**Affiliations:** ^1^ Department of Ultrasound, Affiliated People’s Hospital of Jiangsu University, Zhenjiang, China; ^2^ School of Medicine, Jiangsu University, Zhenjiang, China; ^3^ Department of Pathology, Affiliated People’s Hospital of Jiangsu University, Zhenjiang, China; ^4^ Department of General Surgery, Affiliated People’s Hospital of Jiangsu University, Zhenjiang, China; ^5^ Department of Artificial Intelligence, Julei Technology Company, Wuhan, China; ^6^ Department of Radiology, The Affiliated Hospital, Jiangnan University, Wuxi, China

**Keywords:** nomogram, ultrasound radiomics, papillary thyroid carcinoma, extrathyroidal extension, thyroid neoplasms, ultrasonography

## Abstract

**Purpose:**

To construct a sequence diagram based on radiological and clinical factors for the evaluation of extrathyroidal extension (ETE) in patients with papillary thyroid carcinoma (PTC).

**Materials and Methods:**

Between January 2016 and January 2020, 161 patients with PTC who underwent preoperative ultrasound examination in the Affiliated People’s Hospital of Jiangsu University were enrolled in this retrospective study. According to the pathology results, the enrolled patients were divided into a non-ETE group and an ETE group. All patients were randomly divided into a training cohort (n = 97) and a validation cohort (n = 64). A total of 479 image features of lesion areas in ultrasonic images were extracted. The radiomic signature was developed using least absolute shrinkage and selection operator algorithms after feature selection using the minimum redundancy maximum relevance method. The radiomic nomogram model was established by multivariable logistic regression analysis based on the radiomic signature and clinical risk factors. The discrimination, calibration, and clinical usefulness of the nomogram model were evaluated in the training and validation cohorts.

**Results:**

The radiomic signature consisted of six radiomic features determined in ultrasound images. The radiomic nomogram included the parameters tumor location, radiological ETE diagnosis, and the radiomic signature. Area under the curve (AUC) values confirmed good discrimination of this nomogram in the training cohort [AUC, 0.837; 95% confidence interval (CI), 0.756–0.919] and the validation cohort (AUC, 0.824; 95% CI, 0.723–0.925). The decision curve analysis showed that the radiomic nomogram has good clinical application value.

**Conclusion:**

The newly developed radiomic nomogram model is a noninvasive and reliable tool with high accuracy to predict ETE in patients with PTC.

## Introduction

Papillary thyroid carcinoma (PTC) occurs in 90% of patients with thyroid carcinoma (1–3). PTCs are inert, differentiated cancers with relatively low recurrence and incidence rates. However, some histologic PTC subtypes (high cell count, diffuse sclerosing type, infiltrative type) show aggressive behavior and recurrence with extrathyroidal extension (ETE), vascular invasion, and distant metastasis.

According to the TNM classification of differentiated thyroid cancer by the Eighth Edition of the American Joint Committee on Cancer (AJCC) (2–5), minimal ETE (i.e., T3) refers to a primary tumor of more than 4 cm that is limited to the thyroid gland or has invaded the strap muscles surrounding the thyroid, whereas extensive ETE (i.e., T4) describes the primary tumor invasion of the subcutaneous soft tissue, larynx, trachea, oesophagus, recurrent laryngeal nerve, prevertebral fascia, carotid artery, or mediastinal vessels. Several studies demonstrated that ETE is an independent risk factor for high recurrence and mortality in PTC patients (3, 4). The detection of ETE is also clinically significant regarding the selection of the optimal treatment. PTC surgery mainly involves either total/subtotal thyroidectomy or resection of the affected thyroid lobe and isthmus, but both surgical procedures have no significant effect on postoperative distant metastasis and mortality. Besides, surgical procedures targeting only one thyroid lobe or the isthmus do not only retain some functionality of the thyroid gland but protect also parathyroid functions and prevent injuries of the contralateral laryngeal recurrent nerve. Total/subtotal thyroidectomy is usually suggested for PTC patients with ETE, whereas PTC patients without ETE are treated with resection of the affected thyroid lobe and isthmus. Thus, there is a need for a noninvasive method to evaluate ETE, thereby avoiding total/subtotal thyroidectomy in patients without ETE.

Ultrasound is the most common imaging modality for preoperative PTC diagnosis (6–9), but it is subjective and relies on the experience level of the operator. Computed tomography (CT) has a certain advantage in evaluating whether PTC has invaded neighboring tissue, but CT requires ionizing radiation. Magnetic resonance imaging can improve soft tissue resolution; however, it is expensive and not widely used in the examination of thyroid tumors. Ultrasound radiomics (USR) is a new tool that can extract hundreds of quantitative features from medical images and combine the key features into a radiomic signature, an image-based biomarker, that can be used for cancer diagnosis (10, 11). Some studies demonstrated that ultrasound imaging has great value in the diagnosis of various diseases, as well as the assessment of their prognosis (12–14). It has been reported that the texture features of ultrasound images have good predictive value for cervical lymph node metastasis in PTC (12–15). However, only a few studies analyzed ETE in PTC using USR. Therefore, we developed and validated in this study a USR model for the noninvasive prediction of the preoperative ETE status in PTC.

## Materials and Methods

### Patients

**Figure 1:**
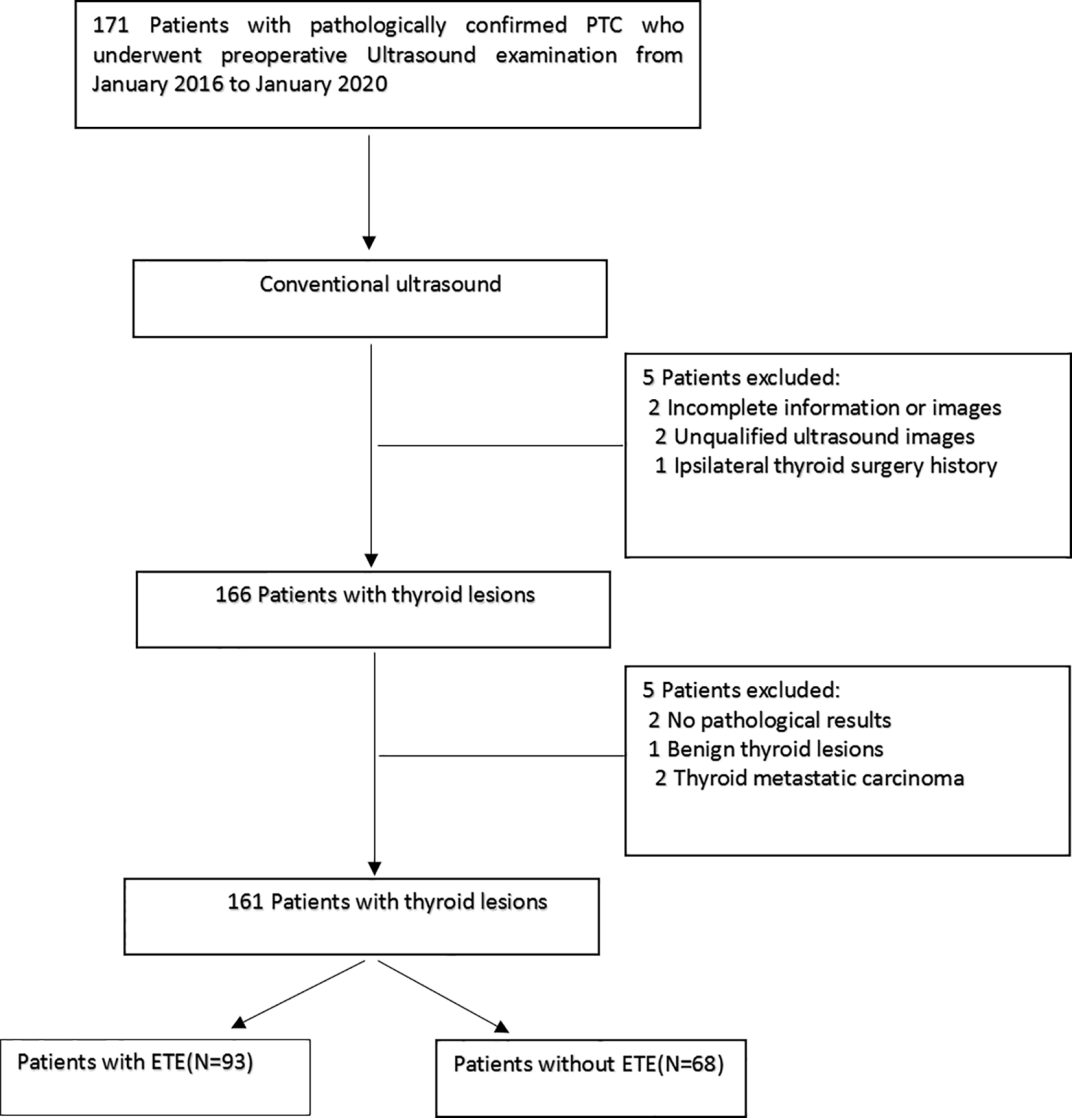
Schematic diagram of the patient selection. PTC, papillary thyroid carcinoma.

This retrospective study was approved by the local ethics committee at the Affiliated People’s Hospital of Jiangsu University, and the requirement for informed consent was waived. Between January 2016 and January 2020, 161 patients of our hospital were retrospectively selected. **Figure 1** shows the enrolment procedure. The inclusion criteria were defined as follows: (1) preoperative thyroid ultrasound examination, providing relevant ultrasound image data, and a PTC diagnosis; (2) the postoperative pathology confirmed the PTC diagnosis; (3) a single unilateral lesion; and (4) no prior thyroidectomy. Exclusion criteria were: (1) the primary tumor was not unequivocally identifiable on the ultrasound image and (2) the maximum diameter of the primary tumor was <5 mm. The clinical and pathological information of the enrolled patients included age, sex, tumor size, tumor position, and tumor location. The AJCC deleted in the eighth edition of the TNM classification the definition of minimal ETE because it was not considered an independent risk factor related to PTC prognosis. However, this concept remains controversial, and some researchers suggest that minimal ETE increases the risk of PTC recurrence (3, 4). Therefore, in this study, minimal ETE and extensive ETE were uniformly classified as ETE.

### Ultrasound Examination

Preoperatively, all patients underwent a routine ultrasound examination, performed by well-trained technicians using a Philips Q5, Philips iU22 (both Healthcare, Eindhoven, the Netherlands) or a GE LOGIQ s8, LOGIQ E20, LOGIQ E9 (GE Medical Systems, American General) ultrasound system with a 5–12 MHz linear array transducer. The patient was placed in a supine position with the pillow removed to lower and slightly recline the head. This exposed the neck region as much as possible to carry out the ultrasound examination of the thyroid and cervical area using longitudinal, horizontal continuous scanning. This allowed the observation of the thyroid tumor size (maximum long axis of the nodule), tumor position (left lobe, right lobe, or isthmus), tumor location (upper, middle, or lower pole), internal echo pattern (uniform, less uniform, or nonuniform), tumor border (clear, less clear, or fuzzy), tumor vascularization (without, rare, or abundant), elastic properties (0 points: tumor tissue color-coded between red and green; 1 point: uniform green tumor; 2 points: tumor mainly between green and blue-green; 3 points: tumor mainly between blue-green and blue; 4 points: uniform blue tumor), and the ETE diagnosis. Preoperative imaging criteria for US diagnosis ETE were as follows: the lesions contact the thyroid capsule >25% or protrude the thyroid capsule to invade the sternum thyroid muscle and the soft tissue around the thyroid gland, the fat space between the trachea, esophagus, trachea and esophagus sulcus, cervical sheath vessels, and the thyroid gland disappears.

### Diagnostic Criteria for Image Analysis

Two radiologists blinded to the clinical and pathological information assessed the ultrasound images, and any disagreement was resolved by agreement. According to the AJCC guidelines (3–7), ETE can be diagnosed when one of the following two criteria exists: (1) >25% of the circumference of the lesion is in contact with the thyroid capsule or the envelope line of the contact between the lesion and the thyroid gland disappears; (2) a tumor of any size exceeds the thyroid capsule and invades the subcutaneous soft tissue, larynx, trachea, oesophagus, recurrent laryngeal nerve, carotid artery, or mediastinal vessels.

### Region of Interest (ROI) Segmentation and Radiomic Feature Extraction

To indicate focal areas within the thyroid gland, ROIs were manually drawn on ultrasound images by one radiologist with 15 years of experience in the diagnosis of thyroid diseases using the software ITK-SNAP (version 3.8.0, http://www.itksnap.org). The ROI was placed on the solid component of the tumor, avoiding necrotic, hemorrhagic, and cystic areas.

To assess the consistency of the ROI placements, 30 patients were randomly selected, and a second physician with 8 years of experience in thyroid ultrasound diagnosis independently placed ROIs on the relevant structures.

From these ROIs on ultrasound images, 479 image features were extracted using PyRadiomics (version 2.2.0, https://github.com/Radiomics/pyradiomics). These features included 18 first-order features, 14 shape features, 16 grey-level run length matrix (GLRLM) features, 16 grey-level size zone matrix (GLSZM) features, 14 grey-level dependence matrix (GLDM) features, 5 neighbourhood grey-tone dependency matrix (NGTDM) features, 24 grey-level co-occurrence matrix (GLCM) features, and 372 features derived from first-order GLCM, GLRLM, GLSZM, GLDM, and NGTDM features using wavelet filter images.

### Feature Selection and Radiomic Signature Construction

**Figure 2:**
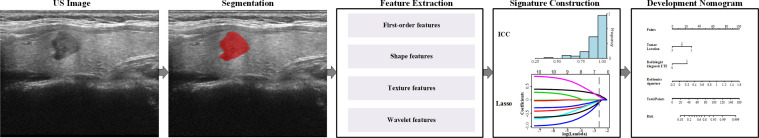
Radiomics workflow in this study.

The consistency of the extracted ROI characteristics was evaluated using the interclass correlation coefficient (ICC). The analysis revealed an ICC of >0.8, demonstrating a good consistency of these characteristics. Next, we used the independent sample t-test or Mann-Whitney U test in the two groups to eliminate nonsignificant features with P-values of >0.05. The minimum redundancy maximum relevance (mRMR) algorithm was employed to assess the relevance and redundancy of the remaining features, and the top 10 features with high relevance and low redundancy were selected for the following analyses. The least absolute shrinkage and selection operator (LASSO) logistic regression model with 10-fold cross-validation was adopted for further feature selection and radiomic signature construction in the training cohort. The radiomic signature was generated by LASSO regression using a linear combination of the selected features with nonzero coefficient weight. Finally, the potential association of the radiomic signature with ETE was evaluated in the training and validation cohorts. **Figure 2** shows the workflow of this study.

### Development of the Ultrasound Radiomic Nomogram

Based on multivariate logistic regression analysis, the clinical model was generated using clinical factors with P-values of **<**0.05. In this model, clinical risk factors such as age, sex, tumor size, tumor position, tumor location, internal echo pattern, tumor border, tumor vascularization, elastic properties, and radiological ETE diagnosis were included. A radiomic nomogram incorporating the radiomic signature and clinical risk factors was developed and used to intelligently predict ETE based on the multivariate analysis in the training cohort (**Figure 2**). For comparison, a clinical model was developed using the independent clinical risk factors alone.

### Performance and Clinical Utility of the Radiomic Nomogram

The radiomic nomogram was evaluated using a calibration curve and the Hosmer-Lemeshow test (a nonsignificant test denotes that the model calibrates perfectly). The nomogram-predicted probability of each patient was calculated according to the nomogram algorithm. The discrimination performance of the radiomic nomogram-predicted probability was evaluated based on the receiver operating characteristic curve, sensitivity, and specificity. Then, the performances of the radiomic nomogram-predicted probabilities were tested in the training and validation cohorts. The decision curve analysis was applied in determining the clinical usefulness of the radiomic nomogram by calculating the net benefits at different threshold values in the combined training and validation cohort (16–18).

#### Histopathological Examination

PTC specimens of paraffin embedding slice, after HE dyeing, by two attending pathologists according to the American Thyroid Association (ATA) published guidelines for the diagnosis and treatment of thyroid cancer malignant degree classification standard, classification under the lens. The tumor is considered to be invasive if one of the following pathological manifestations is present: (1) the vascular or enveloped thyroid gland is invaded by the tumor; or (2) tumor invasion beyond the thyroid, tumor regional metastasis, and distant metastasis.

### Statistical Analysis

Statistical analyses were processed using the R software (version 3.6.1, https://www.r-project.org). Pearson’s chi-square or Fisher’s exact test was used to compare differences for categorical characteristics. The independent sample t-test was performed for continuous factors with normal distribution, whereas the Mann-Whitney U test was used for continuous factors without normal distribution. A two-sided P < 0.05 denoted statistically significant differences.

## Results

### Clinical Characteristics

A total of 161 patients with PTC were enrolled with an average age of 46.09 ± 11.79 years and a male-to-female ratio of 38:123. The pathology excluded ETE in 68 patients and confirmed it in 93 patients. Using stratified sampling, all patients were randomly divided into a training group (n = 97) and a validation group (n = 64). The clinical data of the training group and the validation group are shown in **Table 1**. There was no significant difference between the two groups in pathology and ultrasound image characteristics (all P > 0.05).

**Table 1:** Patient characteristics of the training and validation cohorts.

Characteristic	Training cohort (n = 97)	Validation cohort (n = 64)	P
Age, mean ± SD, years	45.57 ± 11.87	46.89 ± 11.73	0.972
Sex, n			
Female	72	51	0.425
Male	25	13	
Tumor size in ultrasound	10.25 ± 7.99	11.58 ± 8.75	0.089
Tumor location			
Left lobe	45	30	0.991
Right lobe	5	3	
Isthmus	47	31	
Tumor position			
Upper pole	50	31	0.066
Middle pole	32	14	
Inferior pole	15	19	
Internal echo pattern			
Uniform	12	11	0.691
Owe uniform	44	27	
Nonuniform	41	26	
Tumor border			
Clear	35	33	0.254
Less clear	39	21	
Fuzzy	19	9	
Tumor vascularization			
Without	33	19	0.831
Rare	28	33	
Abundant	16	12	
Elastic properties classification			
2	11	7	0.907
3	70	48	
4	16	9	
Radiological ETE diagnosis			
Without ETE	20	22	0.052
With ETE	77	42	

ETE, extrathyroidal extension; SD, standard deviation.

### Radiomic Signature Construction and Diagnostic Validation

**Figure 3:**
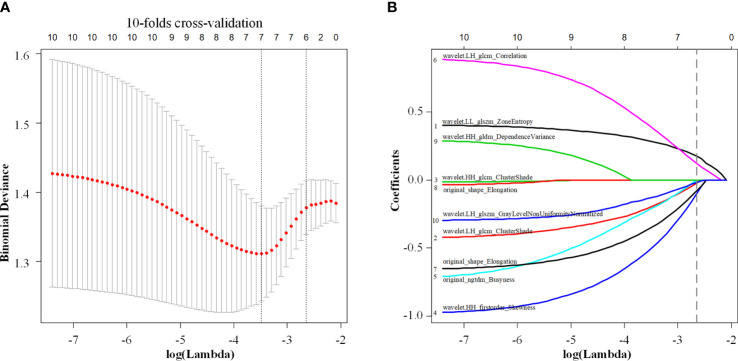
Least absolute shrinkage and selection operator (LASSO) regression with 10-fold cross-validation **(A)** was used to reduce the dimension of the grouping characteristics **(B)**. Six features corresponded to the minimum error.

A total of 479 imaging features were extracted from each greyscale ultrasound image. Of those, 87 image features with ICC values ≤0.8 were deleted, and 256 radiomic features with no statistical significance in the training cohort according to the t-test or Mann-Whitney U test were eliminated. Then, 10 image feature subsets with the best ETE discrimination were screened using the mRMR method. Finally, using LASSO regression and 10-fold cross-validation, six features with nonzero coefficients were selected in the training cohort (**Figure 3**). According to the result of the LASSO regression analysis, the mathematical expression of the radiomic signature was:

$$Radiomic\;signature\;=\;0.335\;+\;0.176\;\times \;LL_{glszm}+\;0.122\;\times \;ngtdm\;-\;0.100\;\times \;HH_{glcm}-\;0.080\;\times \;LH_{glcm\;Corr}-\;0.024\;\times \;LH_{glcm\;Clus}-\;0.016\;\times \;LH_{glszm}$$

th LL_glszm_ being Zone Entropy of the wavelet low frequency filtered image GLSZM, (wavelet.LL_glszm_ZoneEntropy), ngtdm being Busyness of the original image NGTDM (original_ngtdm_Busyness), HH_glcm_ being Cluster Shade of the wavelet high frequency filtered image GLCM (wavelet.HH_glcm_ClusterShade), LH_glcmCorr_ being wavelet low frequency filtered image has correlation of GLCM (wavelet.LH_glcm_Correlation), LH_glcmClus_ being Cluster Shade of GLCM (wavelet.LH_glcm_ClusterShade), and LH_glszm_ being grey-level nonuniformity normalized of GLSZM (wavelet.LH_glszm_GLNUN).

### Development and Validation of the Radiomic Nomogram

The clinical model was built using the variables age, sex, tumor size, tumor position, tumor location, internal echo pattern, tumor border, tumor vascularization, elastic properties, and radiological ETE diagnosis. The univariate logistic regression analysis selected three statistically significant clinical factors, namely tumor size (P = 0.039), tumor position (P < 0.001), and radiological ETE diagnosis (P = 0.002). Multivariate logistic regression analysis was then used to further analyze the influence of these parameters. Because the parameter tumor size was not statistically significant (P = 0.081), this variable was excluded from the model. Thus, tumor position and radiological ETE diagnosis constituted the clinical model for ETE diagnosis in PTC patients (**Table 2** **)**.

**Table 2:** Multivariate logistic regression analysis.

Variable	Clinical model			Radiomic nomogram model		
	β	OR (95% CI)	P	B	OR (95% CI)	P
Cut off	0.671			−0.363		
Tumor location	−1.070	0.343 (0.180–0.653)	0.001	1.065	0.345 (0.172–0.690)	0.003
Radiological ETE diagnosis	1.786	5.964 (1.827–19.46)	0.003	1.645	5.183 (1.470–18.28)	0.011
Radiomic signature	NA	NA	NA	4.130	62.167 (4.760–811.9)	0.002

β, beta coefficient; CI, confidence interval; ETE, extrathyroidal extension; NA, not applicable; OR, odds ratio.

**Figure 4:**
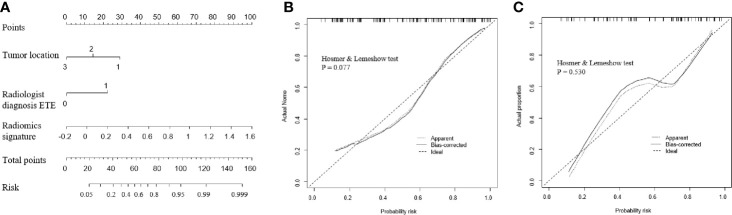
Performance of the radiomic nomogram. **(A)** A nomogram based on the radiomic signature and clinical factors. **(B, C)** Calibration curve of the radiomic nomogram for the training cohort **(B)** and the validation cohort **(C)**. ETE, extrathyroidal extension.

Next, a radiomic nomogram with the parameters tumor location, radiological ETE diagnosis, and radiomic signature was developed. Multivariate logistic regression analysis was used to assess this radiomic nomogram, and all three predictors were statistically significant (**Table 2**, **Figure 4A**). **Table 3** shows the results of the clinical model and the radiomic nomogram model in distinguishing ETE in PTC patients.

**Table 3:** Efficacies of the models predicting ETE in patients with PTC.

	Training cohort			Validation cohort		
	AUC (95% CI)	Sensitivity (95% CI)	Specificity (95% CI)	AUC (95% CI)	Sensitivity (95% CI)	Specificity (95% CI)
Radiomic signature	0.736 (0.633–0.838)	0.679 (0553–0.803)	0.732 (0.585–0.854)	0.760 (0.640–0.879)	0.703 (0.568–0.838)	0.741 (0.556–0.889)
Clinical model	0.768 (0.676–0.860)	0.625 (0.500–0.750)	0.829 (0.707–0.927)	0.741 (0.619–0.863)	0.514 (0.351–0.676)	0.889 (0.778–1.000)
Radiomic nomogram model	0.837 (0.756–0.919)	0.696 (0.571–0.821)	0.927 (0.853–1.000)	0.824 (0.723–0.925)	0.649 (0.486–0.784)	0.741 (0.593–0.889)

AUC, area under the curve; CI, confidence interval; ETE, extrathyroidal extension; PTC, papillary thyroid carcinoma.

In the training cohort, the radiomic nomogram model showed the best discrimination (**Table 3**) with an area under the curve (AUC) value of 0.837 (95% confidence interval [CI]: 0.756–0.919). This AUC value of the radiomic nomogram was higher than that of the clinical model alone (AUC: 0.768, 95% CI: 0.676–0.860; DeLong test, P = 0.031) and the radiomic signature alone (AUC: 0.736, 95% CI: 0.633–0.838; DeLong test, P = 0.024). The radiomic nomogram model also presented the best discrimination (**Table 3** **)** in the validation cohort with an AUC of 0.824 (95% CI: 0.723–0.925), which was higher than that of the clinical model alone (AUC: 0.741, 95% CI: 0.619–0.863; DeLong test, P = 0.012). The calibration curve and the Hosmer-Lemeshow test showed a good calibration in the training set (**Figure 4B**, P = 0.077) and the validation set (**Figure 4C**, P = 0.531). Thus, the nomogram model performed well in both training and validation sets.

**Figure 5:**
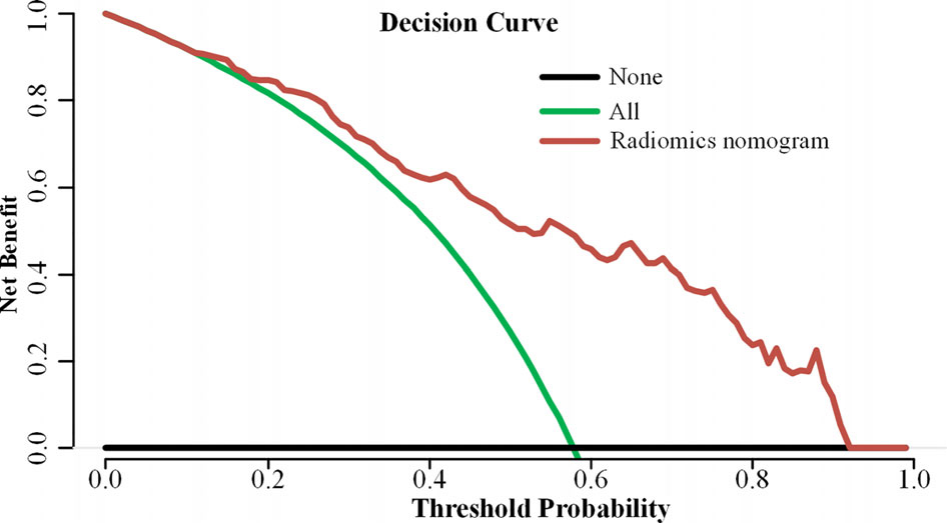
Decision curve analysis of the radiomic nomogram model.

The decision curve analysis demonstrated that the radiomic nomogram provided a high overall net benefit and was more beneficial than either the treat-all or the treat-none strategy (**Figure 5**).

## Discussion

PTC patients with ETE have higher recurrence and mortality rates than those without ETE (1–4). PTC patients with ETE require total/subtotal thyroidectomy; postoperatively, these patients will inevitably be affected by chronic hypothyroidism. Moreover, this procedure is more demanding for the surgeon requiring higher levels of skills, may impair the postoperative parathyroid function, and increases the probability of laryngeal recurrent nerve injury (13–15). Therefore, the accurate diagnosis of ETE before the operation can help the surgeon determine the most suitable surgical plan and reduce the risk of reoperation.

Previous studies showed (6–8) that of the 176 included PTC patients with pathology-confirmed ETE, only 84 patients were accurately diagnosed by ultrasound. Therefore, it is of great importance to improve the accuracy of ultrasound-based ETE diagnosis in PTC. Some studies have suggested that radiomic is of great value in the diagnosis and prognosis assessment of many diseases (16, 19–23). In our study, we used the radiomic nomogram to identify ETE preoperatively. The constructed radiomic nomogram provides an easy-to-use diagnostic and predictive tool, which can prevent unnecessary surgery for patients without ETE. The higher AUC values of the radiomic nomogram indicate that the nomogram including the radiomic signature performs better than the clinical model in diagnosing ETE. The radiomic nomogram model was established using three variables, including the radiomic signature that incorporated six parameters extracted from a large number of image features by data dimension reduction. This model showed a high predictive value for the identification of ETE.

Ultrasound is the preferred imaging modality for the evaluation of PTC (24–27). It can show the degree of PTC contact with the adjacent thyroid capsule, but the diagnostic accuracy is low. Gweon et al. (7) showed that in 79 patients with PTC diagnosed by preoperative ultrasound, the accuracy rates were 60.8% and 66.2% for 2D and 3D ultrasound, respectively. Lee et al. (14) reported that if >50% of the PTC circumference was in contact with the adjacent thyroid capsule, ultrasound had a better AUC than CT (0.674 vs. 0.638, respectively) in the diagnosis of ETE, whereas combined ultrasound and CT had the highest accuracy (sensitivity 92.9%, specificity 70.4%, AUC 0.744). In the present study, the AUC of the radiomic nomogram model for the diagnosis of ETE was significantly higher than that reported by Lee et al. (0.824 vs. 0.744, respectively). This indicates that the newly developed radiomic nomogram model contained more information that is significantly related to ETE but not considered a traditional risk factor. For instance, PTC density and enhanced nonuniformity are characteristics that are difficult to quantify with the human eye. But these features are associated with tissue heterogeneity in PTC. The radiomic nomogram model takes the imaging biomarker PTC heterogeneity, a quantifiable feature related to the degree of malignancy in PTC, into account for the ETE evaluation. Thus, the quantitative nomogram model does not only overcome the subjectivity of the traditional ultrasound imaging diagnosis but also utilizes a lot of information that the naked eye cannot identify, thereby improving the accuracy of the ETE diagnosis.

Our study has several limitations. First, this is a retrospective study, causing a case selection bias that may have affected the study results. Second, our radiomic nomogram model for distinguishing ETE was established and validated in a single hospital. Third, in some PTC cases with unclear boundaries, the tumor was difficult to delineate. These cases were excluded from this study. Most of these cases were PTC with ETE, leading to a certain sample bias in this study. Fourth, the sample size included in this study is not large enough; further multicenter studies with larger sample sizes should be carried out. Forth, since the data in this study are from a single center and the same type of machine, the model will have some robust problems Finally, our radiomic nomogram model only used greyscale ultrasound images, and we will add radiomic characteristics of multimodal ultrasound to the nomogram in the future. In further studies, we will also use elastography and contrast-enhanced ultrasound images, which may contain more radiomic features than conventional 2D images.

In summary, a radiomic nomogram based on clinical risk factors and a radiomic signature was constructed for the prediction of ETE. This nomogram is expected to inform treatment strategies and assist clinical decision-making for a personalized ETE treatment of patients with PTC.

## Data Availability Statement

The original contributions presented in the study are included in the article/supplementary material. Further inquiries can be directed to the corresponding authors.

## Ethics Statement

Written informed consent was obtained from the individuals for the publication of any potentially identifiable images or data included in this article.

## Author Contributions

XW, YR, and EA contributed equally to this study. XW, EA, HS, and GZ contributed to the conception and design of the study. JZ, FX, and XB organized the database. QZ, WL, and YR performed the statistical analysis. XW wrote the first draft of the manuscript. SH and XQ wrote sections of the manuscript. All authors contributed to the article and approved the submitted version.

## Funding

This study was financially supported by National Natural Science Foundation of China (Project No.: 81771848,81971629) and Zhenjiang Commission of Science and Technology (Project No. SH2020046).

## Conflict of Interest

 WL was employed by Julei Technology Company.

The authors declare that the research was conducted in the absence of any commercial or financial relationships that could be construed as a potential conflict of interest.

## Abbreviations

AJCC, American Joint Committee on Cancer; AUC, area under the curve; CI, confidence interval; CT, computed tomography; ETE, extrathyroidal extension; GLCM, gray-level co-occurrence matrix; GLDM, gray-level dependence matrix; GLRLM, gray-level run length matrix; GLSZM, grey-level size zone matrix; ICC, interclass correlation coefficient; LASSO, least absolute shrinkage and selection operator; mRMR, maximum relevance minimum redundancy; NGTDM, neighbourhood grey-tone dependency matrix; PTC, papillary thyroid carcinoma; ROI, region of interest; USR, ultrasound radiomics.
